# Atteinte rénale chez une patiente avec sclérodermie systémique

**DOI:** 10.11604/pamj.2015.21.257.7341

**Published:** 2015-08-07

**Authors:** Faten Frikha, Zouhir Bahloul

**Affiliations:** 1 Service de Médecine Interne, CHU Hédi Chaker, 3029 Sfax, Tunisie

**Keywords:** Sclérodermie systémique, fibrose pulmonaire, atteinte rénale, systemic Sclerodermia, pulmonary fibrosis, renal involvement

## Image en medicine

Madame N.F, âgée de 54 ans, était hospitalisée en 2011 pour toux sèche traînante et une dyspnée d'effort. L'examen des téguments objectivait un visage typique de Sclérodermie systémique (A), une peau indurée, scléreuse au niveau des mains avec un signe de la prière positif (B), des pieds, des avant bras, des jambes, du tronc et du visage. L'examen cardio-pulmonaire révélait des râles crépitants diffus aux deux champs pulmonaires. Sa pression artérielle à 110/70 mmHg. La capillaroscopie révélait un aspect de microangiopathie organique et des mégacapillaires. Le bilan biologique objectivait un discret syndrome inflammatoire, une anémie à 11g/dl et une hypergammaglobulinémie polyclonale à 18g/l. La créatinine sérique était à 139 µmol/l (Clairance de la créatinine à 39 ml/min), la protéinurie des 24 heures était à 0,32 g/jour et le sédiment urinaire était normal. La recherche des AAN était positive à 1/1280 et au typage l'anti -scl70 et l'anti -SSA étaient positifs. La Radiographie du thorax montrait un syndrome interstitiel étendu aux deux poumons (C). Le scanner thoracique trouvait des signes d'atteinte interstitielle fibrosante avancée (D). Une biopsie rénale était réalisée: 3 glomérules dont un était en voie de sclérose, les glomérules restant ne présentaient pas de prolifération endo ni extra capillaire, ils étaient hypertrophiés avec une membrane basale fine. L'atteinte rénale était rattachée à sa sclérodermie systémique. La patiente était mise sous colchicine (1 mg/jour) et un inhibiteur de l'enzyme de conversion.

**Figure 1:**
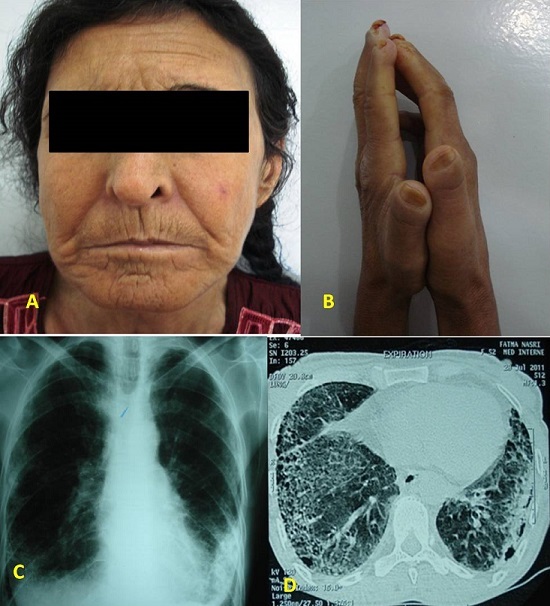
A) aspect de visage sclérodermiforme; B) sclérodactylie avec signe de la « prière » positif; C) radiographie de face objectivant le syndrome interstitiel pulmonaire; D) aspect de fibrose pulmonaire diffuse à la tomodensitométrie pulmonaire

